# Genetics and marker-assisted breeding for sex expression in cucumber

**DOI:** 10.3389/fgene.2023.1180083

**Published:** 2023-06-01

**Authors:** R. K. Dhall, Harleen Kaur, Pooja Manchanda, Eshanee

**Affiliations:** ^1^Department of Vegetable Science, College of Horticulture and Forestry, Punjab Agricultural University, Ludhiana, India; ^2^ School of Agricultural Biotechnology, College of Agriculture, Punjab Agricultural University, Ludhiana, India

**Keywords:** gene expression, genes, gynoecy, inheritance, molecular markers

## Abstract

Cucumber is an important vegetable crop that provides an accessible draft genome, which has significantly expedited research in various fields of molecular genetics. Cucumber breeders have been employing various methodologies to improve the yield and quality of the crop. These methodologies comprise enhancement of disease resistance, use of gynoecious sex types and their association with parthenocarpy, alterations in plant architecture, and enhancement of genetic variability. The genetics of sex expression are a complex trait in cucumbers but are very significant for the genetic improvement of cucumber crop. This review comprises an explanation of the current status of gene(s) involvement and its expression studies, the inheritance of genes, molecular markers, and genetic engineering associated with sex determination, as well as a discussion of the role of ethylene in sex expression and sex-determining genes of the ACS family. There is no doubt that gynoecy is an important trait among all sex forms of cucumber for heterosis breeding, but if it is associated with parthenocarpy, fruit yield can be enhanced to a greater extent under favorable conditions. However, little information is available with regard to parthenocarpy in gynoecious-type cucumber. This review sheds light on the genetics and molecular mapping of sex expression and could be beneficial especially to cucumber breeders and other scientists working on crop improvement via traditional and molecular assistant approaches.

## 1 Introduction

Cucumber (*Cucumis sativus* L.) belongs to the Cucurbitaceae family or vine family; the wild relative *C. sativus* var. *hardwickii* is believed to be its ancestor in India, and its highly diverse wild and cultivated forms are found in the Indian sub-continent (Sebastian et al., 2010). The *Cucumis* genus comprises 66 species, out of which cucumber alone possesses seven pairs of chromosomes with 2n = 14 and a genome size of 367 Mb; the rest have chromosomes as a multiple of 12 (2n = 24) (Weng, 2020).

Cucumber was the first among the various horticultural crops to have a fully accessible draft genome (Huang et al., 2009; Wóycicki et al., 2011; Yang et al., 2012; Qi et al., 2013; Osipowski et al., 2020; Song et al., 2021), which significantly expedited research in crop improvement, breeding processes, and genome editing. The relatively smaller genomic size and its short life span (varying from 4 to 5 months) offer advantages for experiments pertaining to genetic studies in cucumber. The past decades have provided substantial progress in developing bioinformatics resources and facilitating the mapping and cloning of genes and quantitative trait loci (QTLs) of horticultural important traits of cucumber (Weng, 2020; Ma et al., 2022). To date, genomes have been sequenced for six accessions of cucumber, namely, Chinese Long 9930, Gy-14, B10, PI183967, JEF, and KWS (Table 1). The Chinese variety “Chinese Long 9930” (GenBank: GCA_000004075.2) was sequenced by Huang et al. (2009) (updated by Li et al., 2019). The American variety “Gy14” (http://wenglab.horticulture.wisc.edu/) was sequenced by the USDA-ARS (United States Department of Agriculture-Agricultural Research Unit) Vegetable Crops Research Unit, Wisconsin (Cavagnaro et al., 2010). The European Borszczagowski line “B10” (GenBank: GCA_000224045.1) was sequenced by the Polish consortium of cucumber genome sequencing (Wóycicki et al., 2011), and it was updated by Osipowski et al. (2020) with PacBio reads (GenBank: LKUO00000000.2). About 94% of the cucumber genome is covered by B10 and is considered the most comprehensive draft version to date (Osipowski et al., 2020). Recently, Turek et al. (2023) gave more insight into the genome of B10v3 accession by comparing it with the genome of cucumber lines Gy-14 and 9930. Recent advances in genome sequencing have provided stimulating opportunities to accelerate research in cucumber breeding.

**TABLE 1:** Overview of genome sequences of cucumber accessions.

Accession name	Genome size (Mb)	Contig N50 (kb)	Scaffold N50 (Mb)	Anchored (Mb)	Repetitive sequences (%)	Sequencing technology	References
Chinese Long 9930	243.50	19.80	1.14	177.30 (72.80%)	24.00	Sanger and Illumina	Huang et al. (2009)
Chinese Long 9930	226.20	8900.00	11.50	211.00 (93.30%)	36.43	PacBio, 10X Genomics and Hi-C technology	Li et al. (2019)
B10	323.00	23.28	0.32	—	16.09	454 Sanger Celera/Arachne	Wóycicki et al. (2011)
B10	342.29	858.00	—	—	—	Illumina and PacBio	Osipowski et al. (2020)
Gy-14	193.00	—	—	173.10 (89.90%)	—	—	Yang et al. (2012)
PI183967	204.80	119.00	4.20	—	31.10	Illumina	Qi et al. (2013)
JEF	267.7	7362.017	30.57	—	40.50	Illumina and PacBio	Song et al. (2021)
KWS	276.4	8654.608	31.27	—	40.78	Illumina and PacBio	Song et al. (2021)

## 2 Role of genes in sex determination

**FIGURE 1:**
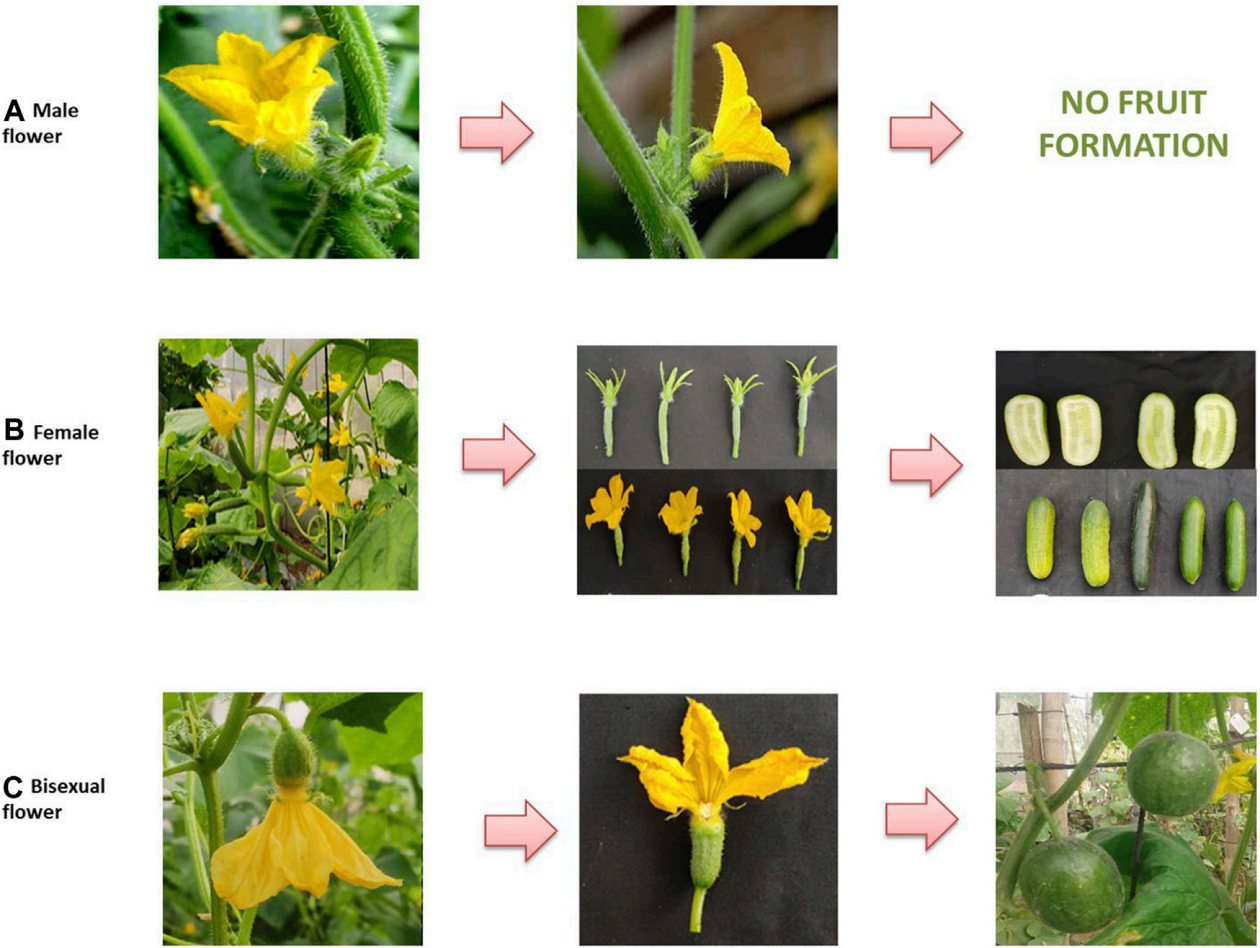
Types of flower sexes and their resulting fruit morphology in cucumber: **(A)** male flowers with the presence of stamens and thin pedicels resulting in no fruit formation; **(B)** female flower with the presence of pistils, ovaries, and thick pedicels resulting in cylindrical fruits; **(C)** bisexual flowers with the presence of both male and female organs and intermediate pedicels resulting in round fruits.

Cucumber plants can have three types of flowers: staminate/male, pistillate/female, and hermaphrodite/bisexual/perfect (Figure 1). Early studies described the role of three genes for sex determination in cucumber: *F* (femaleness), *m* (andromonoecy), and *a* (androecy), belonging to the aminocyclopropane-1-carboxylic acid synthase (*ACS*) gene family (*CsACS1G*, *CsACS2*, *CsACS11*, *and CsACO2*, corresponding to *F*, m, *a*, and *a-1,* respectively) (Kubicki, 1969; Pierce and Wehner, 1990; Trebitsh et al., 1997; Mibus and Tatlioglu, 2004; Li et al., 2009). Thus, the different sex expressions in a cucumber plant may be represented as monoecious (*MMffAA*), gynoecious (*MMFFA/a*), sub-gynoecious (*MMFfA/a*), andromonoecious (*mmffAA*), hermaphroditic (*mmFFA/a*), and androecious (*MMffaa*/*mmffaa*) (Li et al., 2020) (Table 2). The dominant *F* allele is responsible for the gynoecious plant, whereas the recessive *F* allele results in monoecious plants. Linkage of *CsACS1* with the *F* locus was first reported by Trebitsh et al. (1987), who found that the monoecious (*ff*) cucumber plant possessed a single copy of *CsACS1*; however, an additional copy of *CsACS1* (known as *CsACS1G*) was present in the gynoecious (*FF*) plant, which co-segregates with the *F* locus (Table 2). Moreover, the inhibition of *CsACS1G* leading to a monoecious nature confirmed the role of the *F* locus in sex determination (Mibus and Tatlioglu, 2004). Li et al. (2020) reported three genes in gynoecious plants: *CsACS1*, *CsACS1G*, and *CsMYB*. The length of the promoter sequence of *CsACS1G* is longer compared to the promoter sequence of *CsACS1* (Yamasaki et al., 2000). Miao et al. (2011) developed a genetic map comprising microsatellite markers, which located *F* on chromosome 6. The recessive allele *m* is responsible for the development of bisexual flowers, and a pleiotropic gene action for the *m* allele has been observed as *m* alleles also resulted in a spherical fruit shape along with a bisexual flower (Li et al., 2009) (Figure 1). Yamasaki et al. (2001) described the epistatic nature of *M* with respect to *F* and that the plants with the *mmff* genotype are andromonoecious, *mmFF* has bisexual flowers, and *MMFF* has female flowers, whereas *MMff* plants are monoecious. The *M* locus is also known to be the duplicate of *CsACS2* (Li et al., 2009; Table 2), and the level of *CsACS2* may be regulated via ethylene produced by *CsACS1* (Kamachi et al., 2000). *CsACS2* is highly expressed in gynoecious plants as compared to monoecious plants (Yamasaki et al., 2003), and it accumulates in a few flowers of monoecious lines located at specific nodes (Wang et al., 2012). Gene “*a*” is responsible for androecy and is hypostatic to the *F* and has an effect in plants containing only “*ff*”. The “*a*” gene is connected with wild-type *CsACS11* of the *ACS* family (Boualem et al., 2009). Plants with the genetic constitution of *ffaa* are entirely male. The gene was cloned from the rare androecious cucumber variety “EREZ” (Boualem et al., 2015). The recessive “*gy*” locus is due to the mutation in the *WIP* (*CmWIP1–CsWIP1*) gene (Martin et al., 2009; Boualem et al., 2015; Chen et al., 2016), and it resulted in “hard femininity,” which is more stable compared to the “*F*” gene. The “*gy*” locus is linked with the alleged serine/threonine kinase gene *CsPSTK1*, which is involved in ethylene biosynthesis/signaling and sex determination. In the presence of recessive *gy*, *CsPSTK1* encourages ethylene signaling cascade, and regardless of the occurrence of the recessive “*f*” allele, the plant possesses a sufficient amount of ethylene required for pistil promotion. Such an amount of ethylene also induces the M gene, which inhibits stamen development. Thus, a cucumber plant with the *ffMMGy* genotype is monoecious, and the genotype *ffMMgygy* is gynoecious (Pawełkowicz et al., 2012). The *CsWIP1* and *F* genes are located on chromosomes 4 and 6, respectively. The *In-F* (*in*tensive *f*emale) gene was found to result in a high ratio of female flowers in monoecious plants (lacking *F*). Furthermore, a plant with both *F* and *In-F* genes failed to produce male flowers upon treatment with gibberellic acid (Kubicki, 1969). Kubicki (1974) used artificial mutagenesis to identify the *h*ermaphrodite (*h*) allele, which produces bisexual flowers with normal ovaries resembling female flowers in their shape and pollination ability. *tr* (trimonoecious) controls the formation of all types of flowers, i.e., male, female, and bisexual; however, the bisexual flowers have superior ovaries (hypogynous), while female flowers are epigynous (inferior ovaries) (Kubicki, 1969). Cucumber sex determination is also regulated by other genes/QTLs and the application of growth regulators.

**TABLE 2:** Genes involved in different sex forms of cucumber.

Sex form	Genes involved	Sex-determining genes of ACS family (ethylene synthase genes)	Comment
Monoecious	*MMffAA*	*CsACS1*	Plants possessing the recessive *F* allele of *CsACS1* are responsible for monoecious plants. “*A*” (dominant) of *CsACS11* is required for the development of pistillate flowers in monoecious cucumber plants. The dominant *M* inhibits stamen development in monoecious plants, and expression of the dominant *M* of *CsACS2* is low in monoecious plants as compared to gynoecious plants. *M* shows epistasis to *F*. The dominant *Gy* inhibits the expression of *CsPSTK1*, which negatively affects ethylene production
Monoecious	*MMffGy_*	*CsACS11*	
		*CsACS2*	
Gynoecious	*MMFFA/a*	*CsACS1G*	Plants possessing the dominant *F* of *CsACS1G* are responsible for gynoecious plants (dominant femininity, *dF*). *M* shows epistasis to *F*; therefore, when both the genes are dominant (*F*, *M*), gynoecious plants are produced. The dominant *M* of *CsACS2* is high in gynoecious plants as compared to monoecious plants. *A/a* does not show any effect if *F* is dominant as the dominant F masks the effect of *A/a*
Gynoecious	*MMffgygy*	*CsACS1*	The “hard femininity” conferred by *gy* is more stable than that of *F*. The recessive *gy* linked with the *F* has been described as strengthening femaleness in cucumber, and in spite of recessive *f* being present, the plant possesses sufficient ethylene required for the development of pistil
		*CsACS2*	
Sub-gynoecious	*MMFfA/a*	*CsACS1G*	Gene “*a*” is hypostatic to the *F* and has an effect only in *f*. Therefore, *AA* and *aa* do not have any effect because *AA/aa* has an effect only if *ff* is in recessive form. The *M* shows epistasis to *F*. The dominant *M* of *CsACS2* is high in gynoecious plants as compared to sub-gynoecious and monoecious plants
		*CsACS2*	
Hermaphrodite (bisexual)	*mmFFA/a*	*CsACS2*	The recessive *m* is responsible for the development of bisexual flowers. The plant having gene “*m*” is bisexual only if *F* is dominant
		*CsACS1G*	
Androecious	*MMffaa mmffaa*	*CsACS11*	Gene “a” is hypostatic to the *F* and has an effect only in *f*. The “*a*” is connected with the wild-type *CsACS11* of the ACS family
Andromonoecious	*mmffAA*	*CsACS11*	The recessive allele *m* is responsible for the development of bisexual flowers. In the presence of recessive allele *f* along with recessive *m*, male flowers also produce along with bisexual flowers, making the plant andromonoecious
		*CsACS2*	

## 3 Inheritance of sex determination

The type and strength of sex expression are important traits to consider in the commercial production of cucumber as the disparities in this parameter can influence the date of harvest and crop yield.

### 3.1 Gynoecy

Gynoecy is an important genetic mechanism that is exploited for hybrid development. Due to their high yield, earliness, and more female flowers, gynoecy-based cucumber hybrids are achieving popularity among vegetable growers. The phenotypic recognition of the gynoecious trait at the early stages of breeding lines is challenging as the trait is under the influence of environmental factors. Thus, the identification of phenotypically pure gynoecious lines becomes difficult. Therefore, the inheritance of gynoecy needs to be efficiently studied for employing breeding procedures for transferring gynoecious genes into desirable cucumber genotypes. The inheritance of the gynoecious trait reported by different scientists is given in Table 3.

**TABLE 3:** Inheritance of gynoecy and sub-gynoecy in cucumber.

Gene(s)/QTL(s)	References
Gynoecy	
Pair of genes with dominance	Tkachenko (1935)
Single dominant gene (*Acr/F*)	Shifriss (1961); Galun (1962); Mibus and Tatlioglu (2004); Miao et al. (2011); Pati et al. (2015); Jat et al. (2018); Kaur (2019); Win et al. (2019)
Single gene with dominance or incomplete dominance	Lou et al. (2007)
Partial dominance	More and Munger (1987); Perl-Treves et al. (1998); Perl-Treves and Rajagopalan (2018)
Oligogenes with some partial modified genes	Shengjun et al. (2013)
Multiple genes	Kennard et al. (1994); Serquen et al. (1997b); Fazio et al. (2003); Li et al. (2011)
Sub-gyoecy	
Single pair of recessive (*mod-F2*) and incomplete dominant gene (*Mod-F1*)	Chen et al. (2011)
Polygenes and dominance	Win et al. (2019)
Polygenes and semi-dominance	Bu et al. (2016)

Tkachenko (1935) was the first to study the inheritance of gynoecious sex expression in the Japanese variety and reported that “femaleness” was regulated by a pair of genes and this character was dominant to the male character. Galun (1962) and Shifriss (1961) confirmed the monogenic inheritance by discovering the genetic factor *Acr* (later renamed as the *F* locus) responsible for speeding up the number of female flowers in gynoecious lines. The number of female flowers is directly related to the dominant allele (*F*) in a dosage-dependent manner (Shifriss, 1961; Shifriss et al., 1964). The cross of gynoecious × monoecious plants has been studied by various researchers to comprehend the inheritance of gynoecious sex expression, who observed that the gynoecious sex expression is ruled by various factors: the single dominant gene (Jat et al., 2018; Kaur, 2019; Mibus and Tatlioglu, 2004; Miao et al., 2011; Pati et al., 2015; Win et al., 2015), single gene with dominance or incomplete dominance (Lou et al., 2007), partial dominance (More and Munger, 1987; Perl-Treves et al., 1998; Perl-Treves and Rajagopalan, 2018), and oligogenes with modifications in some background genes (Shengjum et al., 2013). In contrast, Li et al. (2011) reported it to be regulated by multiple genes having quantitative inheritance, and they further added the significant influence of seasons on female flowering in diverse germplasms. The polygenic inheritance pattern was also reported by Fazio et al. (2003), Kennard et al. (1994), and Serquen et al. (1997a). Mibus and Tatlioglu (2004) used monoecious (*MMff*) and gynoecious (*MMFF*) cucumber lines for the molecular characterization of *F/f* and observed that the *F/f* locus is linked with *CsACS1G*, concluding that the dominant nature of the *F* allele impacts the amplification of *CsACS1G* in *MMFF* and *MMFf*.

### 3.2 Sub-gynoecy

The inheritance of the sub-gynoecious trait in cucumber was studied by different scientists, and their results are given in Table 3. Chen et al. (2011) used *C. sativus* L. var *sativus* cv. 97-17 and S-2-98 as parents to investigate the inheritance of sub-gynoecy in cucumber plants and observed that both the sub-gynoecious inbred lines were regulated by a single pair of recessive genes and also a single pair of incompletely dominant genes, which could enhance the intensity of femaleness. Moreover, they inherited independently with *F* and *M* and were labelled as *mod-F2* and *Mod-*F1, respectively. Bu et al. (2016) indicated that sub-gynoecy is (semi-) dominant to the monoecy in the F_1_ generation. However, in the BC_1_ and F_2_ populations, continuous variation was observed that indicated the polygenic nature of the sub-gynoecious trait. Similarly, Win et al. (2019) reported the polygenic and dominant nature of the sub-gynoecious trait.

## 4 Molecular markers-assisted breeding for sex expression

### 4.1 Gynoecy

Cucumber’s short genome size makes it ideal for researchers to use molecular breeding techniques to develop cultivars (Table 2). The mapping of gynoecious trait in cucumber and the identification of markers linked to the gynoecious locus are important in marker-assisted backcross breeding for transferring the gynoecious trait to horticulturally desired cucumber genotypes to speed up the cucumber breeding program. Cucumber has a small genetic base and little polymorphism (Kennard et al., 1994). Even though 30 low-resolution linkage maps using RAPDs or AFLPs have been assembled, their dominating nature renders their use unsuitable in MAS (Yuan et al., 2008). However, while such molecular markers could help in identifying gynoecious lines, their dominant traits could not tell the difference between homozygous and heterozygous gynoecious lines. Because heterozygous gynoecious lines may have a less stable gynoecious phenotype than the homozygous lines, an updated molecular marker is still needed to overcome this obstacle. The adoption of DNA markers, particularly SSRs for genes/QTLs that favor mapping, was prompted by whole-genome sequencing of cucumber (Huang et al., 2009). The different molecular markers reported by various scientists for the gynoecious trait in cucumber are given in Table 4. Some polymorphic SSR markers connected to the cucumber gynoecious locus have been described (Zhou et al., 2013; Zhang et al., 2015; Zhu et al., 2016) (Table 5); however, their usefulness is still limited due to the greater genetic distances of the markers from the *F* locus. Fazio et al. (2003) discovered three QTLs linked with the *F* locus in cucumber (two on LG 1, *sex1.1* and *sex1.2,* and one on LG 6, *sex6.1*) and found that the marker CSWCT28 is tightly linked to *sex1.1* (LOD = 13.0), which is mapped 5 cM from the *F* locus on a distal end of linkage group 1. The *F* locus-linked primer pair P2S/P3A was reported to cover the whole precise genomic sequence of the gene *CsACS1G* (Knopf and Trebitsh, 2006). This primer pair spanned the recombination event between the *CsACS1* and *CsBCAT* genes, amplifying the upstream of the *CsACS1G* gene (Wu et al., 2012). The breakpoint of the 30.2-kb duplicated region that designates the *F* locus is marked by the markers Primer F and Primer R (Zhang et al., 2015). Yuan et al. (2008) also identified three QTLs for female flower ratio, out of which two were found on chromosome 2 (*sex2.1* and *sex2.2*) and one on chromosome 6 (*sex6.1*). Guo et al. (2010) sequenced a high number of ESTs from cucumber flower buds of two sex types and discovered differentially expressed genes and potential SSR and SNP markers in these two sex-type flowers. These EST sequences are a useful resource for future functional genomics studies, marker creation, and cucumber breeding. Bommesh et al. (2019) worked on the development and maintenance of gynoecious inbred lines from various cucumber hybrids and made selections in the F_2_ generation and carried them to the F_4_ population, which were validated phenotypically and genotypically through SSR markers. They also identified two polymorphic markers, SSR-02021 and SSR-18718, for the trait.

**TABLE 4:** Molecular markers associated with gynoecious and sub-gynoecious trait in cucumber.

Cross	Mapping population	QTL(s)	Remarks/findings/position	Type of molecular markers	References
		/gene(s)			
G421 (gynoecious) x H-19 (monoecious)	RILs and F_2_	3 QTLs	Three QTLs linked with *F* locus in cucumber (two on linkage group 1, *sex1.1* and *sex1.2*, and one on linkage group 6, *sex 6.1*). QTL (*sex1.1*) is tightly linked to CSWCT28 and CSWCTT14 SSR markers mapped 5 cM f and 0.8 cM from *F* locus on chromosome 1	SSR	Fazio et al. (2003)
240-1-2-2-3-1 (gynoecious) x 3-5-1-3-2-1-1-1-1-2	F_2_	—	Two SSR markers (CSWCT25 and SSR18956) with genetic distance to the gynoecious loci on chromosome 6 were at 7.7 and 6.8 cM, respectively	SSR	Zhou et al. (2013)
(monoecious)					
9110Gt (gynoecious)x 9930 (monoecious)	RILs	—	Markers SSR13251 and CSWCT28 flanked F locus at 1.2 and 1.7 cM, respectively		Miao et al. (2011)
240-1-2-2-3-1 (gynoecious) x 3-5-1-3-2-1-1-1-1-2 (monoecious)	F_2_	—	Markers CSWCT25 and SSR18956 located at genetic distance of 7.7 and 6.8 cM, respectively, from gynoecious loci on chromosome 6	SSR	Shengjun et al., 2013
S-2-98 (Sub-gynoecious) x M95 (monoecious)	F_2_ and BC_1_	3 sub-gynoecious QTLs	*sg3.1* linked to marker SSR13466 on chromosome 3, and *sg6.1* and *sg6.2* linked to markers SSR01308 and SSR02123, respectively, on chromosome 6 for sub-gynoecious trait	SSR	Bu et al. (2016)
PPC-2 (gynoecious) x Pusa Uday (monoecious)	F_2_		SSR 13251 (1.0 cM) and UW020605 (4.5 cM) on chromosome 6 for gynoecy trait	SSR	Jat et al. (2018)
LOSUAS (sub-gynoecious) x BMB (monoecious)	F_2_ and BC_1_	3 sub-gynoecious QTLs	Chromosome 3 (*sg3.1*) and chromosome 1 (*sg1.1* and *sg1.2*): *sg3.1* and *sg1.1* increased femaleness, whereas *sg1.2* decreased femaleness. Bulks of sub-gynoecious and monoecious had 274,377 and 267,386 polymorphic SNPs in F_1_ and BC_1_ populations, respectively	SNP	Win et al. (2019)
Gy-14 (gynoecious) x Pusa Uday (monoecious)	BC_1_F_2_ and BC_1_F_3_	7 gynoecious QTLs	Three major-effect QTLs (*qGyn 5.1*, *qGyn6.5*, and *qGyn6.6* in BC1F2) and two significant QTLs (*qGyn 5.1* and *qGyn6.1* in BC1F3) on chromosomes 5 and 6. Three markers (SSR00233, SSR15516, and SSR13251) were tightly linked to QTLs (*qGyn6.5* and *qGyn 6.6*) at <1.5 cM	SSR	Boopalakrishnan et al. (2020)
G421 (gynoecious) x Pusa Uday (monoecious)	F_2_	—	Markers SSR13251 and SSR15516 closely linked to *F* locus at 1.5 and 4.5 cM, respectively	SSR	Behera et al. (2022)

**TABLE 5:** Polymorphic SSR and InDel primers showing gynoecism on chromosome 6 of cucumber.

S. No.	Primer name	Primer sequence (5′-3′)	Primer length	References
SSR (co-dominant)				
1	CSWCTT14_F	AAA​ATA​TGA​AAC​CCA​TGG​ACA​TGA	24	Fazio et al. (2003)
	CSWCTT14_R	GAT​TAA​ATA​TTG​GGA​ATT​GCT​AA	23	
2	CSWCT28_F	GAA​TTC​AAA​AGC​ATT​TCA​AAA​CTA	24	
	CSWCT28_R	GAA​TTC​AAT​TGG​GTT​TTT​GAA​CCC	24	
3	SSR02123_F	TGG​AAA​ATG​ACA​GCA​ACC​AA	20	
	SSR02123_R	CCA​TTC​TTC​CTT​TCC​ACG​AA	20	
4	SSR11858_F	CCC​TTC​TCT​CTC​CTT​CAA​TCC	21	Cavagnaro et al. (2010)
	SSR11858_R	GTT​TGC​ATG​GTG​AAA​TGT​GG	20	
5	SSR02086_F	AAC​GAC​AGC​GTT​TCC​TCA​CT	20	
	SSR02086_R	GGT​ATA​ATT​GGG​GCG​ATC​CT'	20	
6	CSWCT28_F	GAA​TTC​AAA​AGC​ATT​TCA​AAA​CTA	24	Miao et al. (2011)
	CSWCT28_R	GAA​TTC​AAT​TGG​GTT​TTT​GAA​CCC	24	
7	SSR13251_F	GGT​CAA​TCC​AAA​AGA​GAA​AGC​A	22	
	SSR13251_R	ATC​AAC​ACC​ATT​GAC​GAC​CA	20	
8	SSR15955_F	TTT​GAG​CCT​TGA​GGC​AAA​GT	20	Yang et al. (2012)
	SSR15955_R	GCA​ATT​CAA​CGT​AAT​GGG​CT	20	
9	SSR07248_F	CGA​TTG​GAA​AAT​ATC​GGC​AC	20	
	SSR07248_R	CGA​ATC​GCC​TTC​AGT​TCT​TT	20	
10	SSR21886_F	TCA​GAG​AAA​TGG​AGA​GGG​AAA	21	
	SSR21886_R	CAG​GAT​TTT​TGT​TTG​GGG​AA	20	
11	SSR02021_F	TAA​ACA​TGG​CTT​CCT​CCT​CC	20	
	SSR02021_R	CTC​TCT​TTT​CTC​ACA​CCC​ACA​G	22	
12	UW084131_F	AAG​CCA​AGA​AAA​GGG​TAA​AAA​GA	23	
	UW084131_R	AAA​ATG​TGG​TGG​TTG​GAG​GT	20	
13	UW007281_F	GAG​GAG​GGT​GGT​GAG​TTG​AG	20	
	UW007281_R	CCC​TGT​GGG​TTC​CAC​TCT​AA	20	
14	SSR11798_F	TCC​AAG​CAA​GTT​CAA​TGC​AA	20	
	SSR11798_R	CCC​ATT​TTT​CCT​CTC​CAT​TTC	21	
15	SSR18956_F	CGT​ATG​TAC​GAC​AAA​ATG​TGA​ACA​G	25	Zhou et al. (2013)
	SSR18956_R	TCG​AAA​CCT​CAA​TAC​TTC​TAC​CAA	24	
16	CSWCT25_F	AAA​GAA​ATT​AAG​TCA​ATC​AAA​CCG	24	
	CSWCT25_R	CCC​ACC​AAT​AGT​AAA​ATT​ATA​CAT	24	
17	SSR13251_F	GGT​CAA​TCC​AAA​AGA​GAA​AGC​A	22	Jat et al. (2018)
	SSR13251_R	ATC​AAC​ACC​ATT​GAC​GAC​CA	20	
18	UW020605_F	AAC​AGC​TGT​GCC​CAT​TCT​CT	20	
	UW020605_R	GGT​TTG​AAG​TCC​GCC​ATT​AG	20	
19	SSR15516_F	TGA​GGG​TTT​AAA​AGA​AAA​AGG​TG	23	Kaur (2019)
	SSR15516_R	GCC​AAT​TCC​CCA​ATT​CTT​AAT	21	
InDel (co-dominant)				
1	*Cs-BCAT*_F	CAT​TGT​GTG​AAT​GAA​GAC​AAG 3′	21	Win et al., 2015
	*Cs-BCAT*_R	TTC​AAC​GCA​AAA​CCT​TCA​TC 3′	20	

Miao et al. (2011) generated a linkage map with 148 recombinant inbred lines (RILs) and microsatellite markers derived from a cross between the two inbred lines 9110Gt and 9930. It was known that chromosome 6 harbors the gene of gynoecious sex expression (*F*). Furthermore, two markers flanking the *F* locus, CSWCT28 and SSR13251, were identified at locations 1.2 and 1.7 cM away from the *F* locus, respectively. They also revealed three significant QTLs for the node at which the first female flower appears in the RILs derived from monoecious (*ff*) x gynoecious (*FF*) cucumber lines. Chen et al. (2011) discovered two sub-gynoecious loci, *mod-F2* and *Mod-F1*, which were regulated by a single pair of recessive genes and incompletely dominant genes, respectively. Zhang et al. (2015) reported a copy number variation (CNV) containing four genes that define the *F* locus and produce gynoecious cucumber plants with exclusively female flowers and fruit at practically every node. A recent 30.2-kb duplication at a meiotically unstable site resulted in the CNV, which was likely caused by micro-homology-mediated break-induced replication. Jat et al. (2018) investigated molecular mapping of the gynoecious (*F*) locus and discovered markers SSR13251 and UW020605, which were tightly linked to the *F* locus in cucumber, with stretch distances of 1.0 and 4.5 cM, respectively. Boopalakrishnan et al. (2020) detected seven gynoecious QTLs on chromosomes 5 and 6 in the BC_1_F_2_ population of cucumber, of which the three major-effect QTLs were *qGyn 5.1*, *qGyn 6.5*, and *qGyn 6.6*, and the markers SSR00233, SSR15516, and SSR13251 flanked the QTLs *qGyn6.5* and *qGyn6.6*. In this way, understanding the genes that control floral traits could aid vegetable breeders in developing new, more stable, and climate-resilient gynoecious lines to make hybrid seed production easier.

### 4.2 Sub-gynoecy

A sub-gynoecy nature demonstrates a high level of female character, which might be useful as a substitute for a gynoecious nature under regular or limited development settings. The different molecular markers reported by various scientists for the sub-gynoecious trait in cucumber are given in Table 4. Bu et al. (2016) developed F_2_ and BC_1_ populations from S-2-98 (sub-gynoecious) and M95 (monoecious) to explore sub-gynoecy quantitatively and discovered three QTLs (*sg3.1*, *sg6.1*, and *sg6.2*) on chromosomes 3 and 6 with tightly linked markers SSR13466, SSR01308, and SSR02123, respectively, for the sub-gynoecious trait. The peak marker for *sex6.1* given by Fazio et al. (2003) was located at a genetic distance of 63.8 cM from marker SSR02123 and was tightly linked to *sg6.2* on chromosome 6. Another major-effect QTL (*sg3.1*) comprising a 799 kb genomic region accounted for 54.6% of phenotypic variation. Win et al. (2019) utilized sub-gynoecious “LOSUAS” and monoecious “BMB” and developed the F_2_ and BC_1_ populations to identify the quantitative nature of sub-gynoecy in cucumbers. They identified one major-effect QTL on chromosome 3 (*sg3.1*) and two minor QTLs on the short and long arms of chromosome 1 (*sg1.1* and *sg1.2*). The major- and minor-effect QTLs were able to increase and decrease the degree of femaleness, respectively. A gene responsible for encoding GA20-oxidase was designated as candidate gene for the *sgy3.1* locus.

Phenotypically, the sub-gynoecious nature, which is independent of the *F* gene but bears similarity to the one that is heterozygous at the *F locus* (*Ff*), usually initiates with male flowers development at the first few nodes (1–10) followed by continuous female flowers at later nodes. When QTL mapping for the percentage of female flowers was tested using populations derived from gynoecious (*FF*) × monoecious (*ff*) crosses, the major-effect QTL was found in accordance with the *F* locus (Fazio et al., 2003; Yuan et al., 2008). These observations suggested that multiple genetic factors are responsible for controlling the percentage of female flowers, although *F* and *sgy3.1* played a pivotal role in gynoecious and sub-gynoecious plants, respectively.

## 5 Gene expression studies and transcriptome analysis

The plant transcriptome provides essential information about the gene expression level related to plant growth, cellular differentiation, and morphogenesis. The expression profiles of the genes vary temporally and spatially, which reflects the important role of the transcriptome in plant development. Various techniques used to study gene expression include transcriptome profiling, suppression subtractive hybridization (SSH), microarray analysis, cDNA-amplified fragment length polymorphism, and reverse transcription polymerase chain reaction (Wu et al., 2010). In relation to sex differentiation in cucumber, Wu et al. (2010) identified transcription factors including *rf2* (*translation releasing factor 2*), *PnLHY2, EREBP-9* (*ethylene-responsive transcription factor*), *MYB*, *IF-2* (*translation initiation factor*), *BHLH*, *BTF3*, *WAKY*, *DREB3*, and *TGA2*, which were differentially expressed in gynoecious mutants and monoecious inbred lines. The spatial expression of *EREBP-9* was observed specifically in the ovule at the development of the first phase. *EREBP-9* transcription factors have a DNA-binding domain that binds to *APETALA2* (Riechmann et al., 1998).

Various studies on transcriptome analysis have identified differentially expressed genes in gynoecious and monoecious cucumber lines. Transcriptome sequencing and comparative analysis of expressed sequence tags and mRNA sequences in gynoecious and hermaphrodite cucumber plants identified two zinc-finger transcription factors belonging to the *CmWIP1* family that were highly expressed in gynoecious and hermaphrodite plants (Guo et al., 2010). *CmWIP1* expression causes carpel abortion, which results in the development of unisexual male flowers (Martin et al., 2009). Moreover, a gene belonging to the BZR1-BES1 family (brassinosteroid signaling pathway gene family) was highly expressed in hermaphrodite flowers (Guo et al., 2010). Shoot tips of gynoecious cucumbers treated with gibberellic acid exhibited high expression of genes *SAUR32*, *WRKY41*, *CYP450*, *MYB21*, *NAC2*, *NAC72*, *SAUR21*, *1AA13*, *CKX1*, and *ETR1*, as identified through transcriptome and qRT-PCR analysis (Zhang et al., 2017; Zhang et al., 2019).

Pan et al. (2018) performed the transcriptome analysis of apical shoots of near-isogenic lines of cucumber, which included gynoecious, monoecious, and hermaphrodite, and revealed that B-class floral homeotic genes *CsPI* and *CsAP3* were highly expressed in monoecious lines but suppressed in gynoecious lines. Meanwhile, the C-class floral homeotic gene *CsAG2* was highly expressed in gynoecious lines. They also reported the gene *HECATE* showing 45 times more expression in gynoecious lines as compared to monoecious and hermaphrodite lines. The gene is known to be involved in auxin-mediated gynoecy in *Arabidopsis* (Gremski et al., 2007). Other genes that were found to be highly expressed in gynoecious lines included *CsIAA29, ETR1,* and gene encoding histidine phosphotransfer protein. Pawelkowicz et al. (2019) identified the genes expressing in gynoecious and monoecious cucumbers and grouped them into various categories including transcription factors (*APETALA*, *SEPALLATA*, *AGAMOUS*, *SEEDSTICK*, *HAT5*, *WUS*, *KNOX STM SHOOT MERISTEMLESS*, and *PISTILLATA*), genes involved in processes of hormones (*ETR*, *ERS*, *EIN*, *MTO1*, *CKX7*, *GAMYB1*, *ACS1G*, and *BRI1*), metal ions (genes encoding metal oxides), cell wall synthesis, cytochromes (*BCS1*, *BAS1*, *CYP75B1*, *CYP71B2*, *CYP716A1*, and *CYP78A10*), sugar and lipid metabolism, and ubiquitination processes.

Sex determination in cucumber is also influenced by light as exposure of cucumber seedlings to red and blue light in the ratio 2:1 resulted in the development of female flowers (Song et al., 2018). The transcriptome analysis revealed that the genes involved in hormone signaling pathways constituted the majority of differentially expressed genes in seedlings treated with a higher proportion of red light. The development of female flowers in response to blue light was found to be regulated by the upregulation of genes involved in auxin and abscisic acid signal transduction pathways, photosynthesis, starch, and sucrose metabolism (Zhou et al., 2018). Wang et al. (2018) performed a transcriptome analysis of the monoecious cucumber cultivar “C09-123,” grown under conditions of low (26°C/12°C) and high (26°C/24°C) temperatures, to study their effect on sex differentiation and observed that male flower development was induced by high night temperatures governed by the genes *AP3*, *PI*, *SEP1*, and *GASA* (gibberellin-regulated family protein).

## 6 Genetic engineering for sex expression

Genetic engineering involves the cloning of a gene of interest and its delivery to the required plant to achieve a specific phenotype with stable expression. The technique has been successfully employed in cucumber to achieve desired mutants. In cucumbers, the genetic transformation efficiency ranges from 1% to about 23% (Feng et al., 2023). The foremost study of CRISPR/Cas9 gene editing in cucumber was carried out to develop transgenic-free cucumber plants with resistance against viruses (Chandrasekaran et al., 2016). However, little has been explored with regard to sex expression as the trait is highly complex and regulated by environmental factors. Recently, the optimization of CRISPR/Cas9 system has been achieved by employing a stronger CsU6 promoter and a GFP tag in the technique, which makes it possible to select between transformed and transgene-free mutants in the progeny (Feng et al., 2019). Transgene-free cucumber plants with editing in *CsWIP1*, *CsVFB1*, *CsMLO8*, and *CsGAD1* have also been developed. The first study to report knocking off *CsWIP1* to increase femaleness in cucumbers has laid the pathway to achieve a gynoecious character by using CRISPR/Cas9-mediated mutagenesis (Hu et al., 2017). After changing *CmWIP1* (carpel development inhibitor) to *CsWIP1*, a significant increase in the formation of gynoecious inbred lines in cucumber was noticed, which might be used for hybrid seed production. Zhang et al. (2021) successfully introduced the complete genomic region of *ACS1G* into monoecious plants to transform them into gynoecious lines. These studies have helped to establish protocols for genetic engineering targeted at gynoecious sex expression in cucumber.

## 7 Conclusion

Cucumber was the foremost among the various horticultural crops to have a fully accessible draft genome in three varieties (Chinese Long 9930, Gy14, and B10) due to its small genome size (367 Mb) and short life span (varying from 4 to 5 months), which offered substantial progress in developing bioinformatics resources, facilitating the mapping and cloning of genes, and discovering QTLs of important horticultural traits in cucumber. The inheritance of the gynoecious sex expression has been studied by various researchers, who have reported that the gynoecious sex expression is governed by various factors: the single dominant gene, a single gene with dominance or incomplete dominance, partial dominance, oligogenes with some background genes modified, and polygenes. There is no doubt that gynoecious sex expression is genetically controlled, but environmental conditions such as longer days and higher temperatures induce male flower formation, whereas female flower development is aided by shorter days and lower temperatures. The interaction of genes and the environment has immediate consequences, such as the instability of gynoecious sex expression in cucumber. The mapping of gynoecy in cucumber and identification of markers linked to the gynoecious locus is important in marker-assisted backcross breeding for transferring the gynoecious trait to horticulturally desirable cucumber genotypes in order to speed up the cucumber breeding program. However, some SSR markers connected to the cucumber gynoecious locus have been developed; nonetheless, their usefulness is still limited due to the greater genetic distances from the markers to the F locus. Different scientists have reported the QTLs linked with the *F* locus on different chromosomes (1, 3, 5, 6), which creates confusion. Therefore, there is a need to identify the QTL(s) linked with the genes controlling the gynoecious trait. Moreover, genetic engineering can be further used to knock out the genes responsible for male flower induction in the monoecious lines and can help in the development of stable gynoecious lines.
